# Whole genome expression analysis within the angiotensin II-apolipoprotein E deficient mouse model of abdominal aortic aneurysm

**DOI:** 10.1186/1471-2164-10-298

**Published:** 2009-07-06

**Authors:** Catherine Rush, Moses Nyara, Joseph V Moxon, Alexandra Trollope, Bradford Cullen, Jonathan Golledge

**Affiliations:** 1 The Vascular Biology Unit, School of Medicine, James Cook University, Townsville, Australia

## Abstract

**Background:**

An animal model commonly used to investigate pathways and potential therapeutic interventions relevant to abdominal aortic aneurysm (AAA) involves subcutaneous infusion of angiotensin II within the apolipoprotein E deficient mouse. The aim of this study was to investigate genes differentially expressed in aneurysms forming within this mouse model in order to assess the relevance of this model to human AAA.

**Results:**

Using microarrays we identified genes relevant to aneurysm formation within apolipoprotein E deficient mice. Firstly we investigated genes differentially expressed in the aneurysm prone segment of the suprarenal aorta in these mice. Secondly we investigated genes that were differentially expressed in the aortas of mice developing aneurysms relative to those that did not develop aneurysms in response to angiotensin II infusion. Our findings suggest that a host of inflammation and extracellular matrix remodelling pathways are upregulated within the aorta in mice developing aneurysms. Kyoto Encyclopedia of Genes and Genome categories enriched in the aortas of mice with aneurysms included cytokine-cytokine receptor interaction, leukocyte transendothelial migration, natural killer cell mediated cytotoxicity and hematopoietic cell lineage. Genes associated with extracellular matrix remodelling, such as a range of matrix metalloproteinases were also differentially expressed in relation to aneurysm formation.

**Conclusion:**

This study is the first report describing whole genome expression arrays in the apolipoprotein E deficient mice in relation to aneurysm formation. The findings suggest that the pathways believed to be critical in human AAA are also relevant to aneurysm formation in this mouse model. The findings therefore support the value of this model to investigate interventions and mechanisms of human AAA.

## Background

Abdominal aortic aneurysm (AAA) affects approximately 5% of men and 1% of women aged >60 years [1]. The principal concern with the condition is aortic rupture which is frequently fatal. Currently the only treatment option for AAA is surgery and approximately 25,000 aortic repairs are performed annually in the USA [2]. AAA accounts for approximately 15,000 deaths annually in the USA despite the increasing numbers of elective aortic repairs [2,3]. There is increasing interest in using animal models of AAA to investigate mechanisms important in aneurysm development and progression in order to develop new non-interventional treatments and better ways of monitoring disease progression [4]. Currently rodent models of AAA are most commonly employed, particularly through infusion of angiotensin II in hyperlipidaemic mice, such as apolipoprotein E deficient (ApoE^-/-^) animals [5-7]. This mouse model has a number of similarities to human AAA such as the preponderance of aneurysm formation in males and the focal nature of aortic dilatation [5,6]. The model also displays a number of differences from human AAA, for example aneurysms commonly involve the suprarenal aorta, dissection is an important histological finding and the infrarenal aorta is rarely if ever affected [7]. In contrast the infrarenal aorta is the most common site of AAA in humans. These and other disparities between this mouse model and human AAA stimulated us to investigate the gene expression profile of aneurysms in the ApoE^-/- ^mouse model. The aim of the current study was to identify genes and pathways associated with aneurysm formation in the angiotensin II infused ApoE^-/- ^mouse model. We used three approaches. Firstly we aimed to identify genes which might underlie the protection of the infrarenal aorta from aneurysm formation in ApoE^-/- ^mice. Secondly we aimed to identify genes which were differentially expressed within the aortas of mice developing aneurysms in comparison to those that did not. Finally we selectively examined whether the differences in gene expression associated with aneurysm formation translated into similar changes in protein expression. The design of these studies is illustrated in Table 1. Our findings suggest the importance of chemokines, pro-inflammatory cytokines, leukocyte transendothelial migration mechanisms, a number of different signalling pathways (such as the Janus kinase pathway) and proteolytic mechanisms in aneurysm formation in these mice supporting the relevance of this model to human AAA [8-10].

**Table 1:** Summary of the included studies.

**Study**	**Comparison**	**Design**	**Method**	**Mice**	**Genes identified**
1	Paired supra and infrarenal aortas	Pooled samples	Codelink mouse whole genome bioarray (36227 genes)	10	Differentially expressed between AAA prone supra and infrarenal aorta

2	Whole aortas of mice exposed to angiotensin II that did and did not develop aneurysms and saline infused controls	Individual mice	Illumina mouse sentrix 6 microarray (46,628 genes).	18	Differentially expressed between the 3 groups revealing aneurysm-associated and "protective" genes

3	Suprarenal aortas of mice exposed to angiotensin II that did and did not develop aneurysms	Individual mice	ELISA and IHC	18*	Validate findings from the microarray in study 2 for Tnfrsf11b, Tgfb1, and inflammatory cell markers.

* 10 further mice died and were excluded from this study.

## Results

### Comparison of gene expression in aneurysm prone suprarenal compared to aneurysm resistant infrarenal aorta

A total of 26,522 transcripts (73% of the reference list) were expressed above background and compared between segments. A total of 304 transcripts were differentially expressed (1.5 fold, p < 0.05) between supra and infrarenal aortic segments (53 upregulated within the suprarenal aorta and 251 upregulated within the infrarenal aorta). A full list of transcripts is given in Additional File 1 and examples of potentially relevant genes shown in Table 2. A number of these genes may be relevant to the predilection of the suprarenal aorta to aneurysm formation based on knowledge of their functional importance or their previous identification in human AAA association studies [9]. Ucn3 for example, which was downregulated in the suprarenal aorta, has been demonstrated to antagonize angiotensin induced production of oxygen derived free radicals *in vitro *and play a vascular protective role in animal models [11,12]. Pik3ap1, Dapk1 and Cd59 have previously been identified to be upregulated within human AAA biopsies [9]. Since vascular smooth muscle apoptosis and inflammation have been demonstrated to be important mechanisms in human AAA, upregulation of Dapk1 and Cd59 may be relevant to the predilection of the suprarenal aorta to aneurysm formation in ApoE^-/- ^mice [8]. Perhaps more important to the predilection of the suprarenal aorta to aneurysm formation are the downregulation of a number of genes coding for structural extracellular matrix proteins including Lama3, Myo18b and the cytoskeletal protein Tuba8 (Table 2). Lama3 has been identified to be downregulated within human AAA biopsies [9].

**Table 2:** Examples of genes differentially expressed between paired supra and infrarenal aortas.

**Symbol**	**Gene ID**	**Gene Name**	Mean expression ratio*	**P value**
Adprhl1	234072	ADP-ribosylhydrolase like 1	3.11	0.015

Lgtn	16865	Ligatin	2.91	0.041

Fabp3	14077	Fatty acid binding protein 3	1.86	0.043

Pik3ap1†	83490	phosphoinositide-3-kinase adaptor protein 1	1.85	0.030

Dapk1†	69635	Death associated protein kinase 1 (Dapk1)	1.73	0.028

Tuba8	53857	tubulin, alpha 8	1.68	0.045

Cd59a†	12509	CD59a antigen	1.68	0.007

Cxcl13	55985	chemokine (C-X-C motif) ligand 13	1.56	0.021

Tgfbr3†	21814	Transforming growth factor, beta receptor III	1.53	0.030

Myo18b	74376	myosin XVIIIb	0.59	0.006

Ucn3	83428	urocortin 3	0.57	0.022

Srd5a2	94224	steroid 5 alpha-reductase 2	0.56	0.029

Dsc3	13507	desmocollin 3	0.52	0.037

Lama3†	16774	laminin, alpha 3	0.52	0.025

Trat1†	77647	T cell receptor associated transmembrane adaptor 1	0.51	0.008

Shbg	20415	sex hormone binding globulin	0.42	0.034

Akr1b7	11997	aldo-keto reductase family 1, member B7	0.24	< 0.001

Shown are a subset of the 304 gene transcripts that were differentially expressed in suprarenal compared to infrarenal aortic segments (1.5-fold, uncorrected p value < 0.05 calculated with paired t-test; for a full list see Additional File 1). The genes were selected from the full list based on the potential relevance to AAA. Genes highlighted with † have previously been identified as associated with human AAA in a whole genome expression study [9]. *Ratio of relative gene expression in supra compared to infrarenal aortic segments from 10 paired samples (5 pairs of arrays due to pooling of 2 aortic segments for each analysis).

### Genes expressed in aortas from mice with aneurysms compared to those without aneurysms and controls

**Figure 1:**
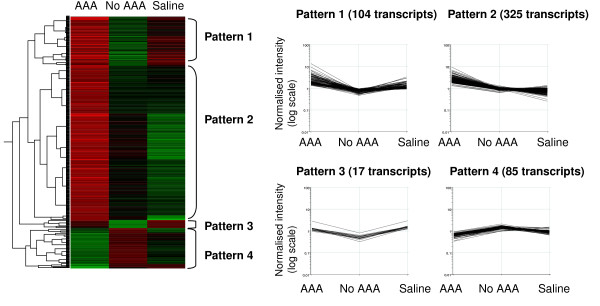
**Heatmap of 531 genes differentially expressed in the aortas of angiotensin II treated mice that developed aneurysms (n = 5) versus those that did not develop aneurysms (n = 7)**. A complete list of the genes used for this analysis is provided in Additional File 3. Expression in the saline controls (n = 6) for each of these genes is also shown. Each line in the heatmap represents a gene with mean upregulated *(red) *or downregulated *(green) *expression for the three groups. Black colour denotes no change in expression. Different patterns of gene expression are indicated as Patterns 1–4 and associated gene lists are provided in Additional Files 6, 7, 8 and 9 and Table 4. A Volcano plot (two fold change, p < 0.05) comparing the aneurysm group with aneurysm resistant group was used to select genes for this analysis.

Maximum aortic diameters measured in the aortic arch, thoracic, suprarenal and infrarenal aortas in saline controls and mice exposed to angiotensin II that did and did not develop aneurysms are shown in Table 3 for animals used in the microarray experiment (study 2). Aortic diameters were similar between non-aneurysmal angiotensin II perfused mice and saline controls. We used Volcano Plots to identify differentially expressed genes between groups using our pre-defined criteria, i.e. 2-fold differential expression and uncorrected p value < 0.05 by unpaired t-test. The numbers of transcripts differentially expressed in comparison between the 3 groups of mice are shown in Additional File 2. 531 transcripts were differentially expressed (two fold change, p < 0.05) between aortas of mice exposed to angiotensin II which developed aneurysms and those that did not develop aneurysms (aneurysm resistant) (446 up and 85 downregulated, a full list is given in Additional File 3). 1196 transcripts were differentially expressed (two fold change, p < 0.05) between aortas of mice exposed to angiotensin II which developed aneurysms and saline controls (749 up and 447 downregulated, Additional File 4). 654 transcripts were differentially expressed (two fold change, p < 0.05) between aortas of mice exposed to angiotensin II which did not develop aneurysms and saline controls (128 up and 526 downregulated, Additional File 5). To further analyse these results, we concentrated on the transcripts differentially expressed between the aortas of mice that had been exposed to angiotensin II and had or had not developed AAAs (n = 531 listed in Additional File 3). We considered that genes identified by this analysis would be most likely to represent those relevant to aneurysm formation or resistance rather than simply reflecting the effects of angiotensin II. We also wanted to incorporate some of the findings relevant to the saline control group as they enabled us to have a measure of the baseline level of expression of different genes. This group allowed us to better postulate genes upregulated in the AAA resistant aortas, and thus of potential protective value against AAA. We carried out hierachical clustering analysis incorporating expression levels from all three groups of mice for the 531 transcripts that were differentially expressed in angiotensin II treated mice with and without aneurysms. This analysis is illustrated in terms of a heatmap, with a gene tree showing clustering of individual transcripts, in Figure 1. Each line in the heatmap represents a transcript with mean upregulated *(red) *or downregulated *(green) *in expression within the three groups. No change in expression is indicated in black. Hierachical clustering analysis indicated four main patterns of transcript expression (Figure 1, patterns 1–4). Transcripts grouped in pattern 1 (104 transcripts) showed decreased expression in aneurysm resistant aortas relative to other groups and included genes such as *Ccl5*, and *Mmp9 *which have been noted to be upregulated in human AAA biopsies (Additional File 6) [9]. Pattern 2 transcripts (325 transcripts) showed increased expression in aneurysms and included many genes associated with inflammation including matrix metalloproteinase encoding genes (e.g. *Mmp2*), genes encoding markers of inflammatory cells (*Cd14, Cd68*) and many cytokine and chemokine genes (e.g. *Il1b, Il6, Ccl4, Ccl7, Ccl8, Ccl19, MMP2*) (Table 4 shows a selected list and Additional File 7 a full list). Many of these genes, particularly those related to leukocyte chemotaxis, have previously been identified as upregulated within human AAA biopsies (highlighted in Table 4) [9,10]. Pattern 3 transcripts (17 transcripts) were downregulated in the aortas of mice resistant to aneurysm formation. The difference between pattern 1 and pattern 3 transcripts is that in pattern 1, genes are most highly expressed in the AAA group, whereas pattern 3 genes are, in general, more highly expressed in the saline group. This can be clearly seen on the heatmap (Figure 1) where pattern 1 genes in the AAA group are mainly coloured red compared to red/black in saline group, whereas pattern 3 genes are red/black in the AAA group and red in the saline group. Although the clustering algorithim has separated these groups of genes, we believe that both pattern 1 and pattern 3 genes can be considered "pathogenic". Additional File 8 shows transcripts (out of those in pattern 3) that were associated with known genes and were significantly downregulated (2-fold, p < 0.05) with respect to both saline controls and mice that develop aneurysms. Two of these "pathogenic" pattern 3 genes, namely *Apoc1 *(Apolipoprotein C-I) and *Stxbp2 *(syntaxin binding protein 2), have been previously demonstrated to be upregulated within biopsies of human AAA [9]. Pattern 4 transcripts (85 transcripts) were upregulated in mice resistant to aneurysm formation (examples shown in Table 5 and a full list in Additional File 9) and we considered that upregulation of these may be "protective" for aneurysm formation. It is interesting to note that a number of the genes identified in pattern 4 have been previously shown to be downregulated within human AAA biopsies supporting their potential protective role (see Table 5 for examples) [9].

**Table 3:** Maximum aortic diameters of mice exposed to saline or angiotensin II that did or did not develop macroscopic aneurysms used in the microarray experiment (study 2).

**Group**	**Diameter (mm)**				
	
	**Arch**	**Thoracic**	**Suprarenal**	**Infrarenal**	**Average Maximum^#^**
Angiotensin II and aortic aneurysm (n = 5)	1.61 ± 0.18	1.11 ± 0.15	2.06 ± 0.48	0.72 ± 0.18	1.38 ± 0.17

Angiotensin II and no aortic aneurysm (n = 7)	1.34 ± 0.10	0.9 ± 0.10	1.13 ± 0.19	0.65 ± 0.09	1.0 ± 0.07

Saline controls (n = 6)	1.35 ± 0.11	1.16 ± 0.11	0.97 ± 0.08	0.67 ± 0.06	1.0 ± 0.06

Data represent mean values ± standard deviation; #calculated by averaging maximum diameters for all 4 aorta regions in each mouse.

**Table 4:** Examples of genes upregulated in the aortas of mice with aneurysms.

**Gene symbol**	**Gene ID**	**Description; synonym**	**Fold increase cf no AAA**	**P value**	**Fold increase cf saline**	**P value**
**Chemokines/receptors**						

*Cxcl10*	15945	chemokine (C-X-C motif) ligand 10; IP-10	2.766	0.0284	2.487	0.0374

*Cxcl12*	20315	chemokine (C-X-C motif) ligand 12; SDF-1	3.022	0.0121	3.03	0.00358

*Cxcl14*	57266	chemokine (C-X-C motif) ligand 14; MIP-2g	3.812	0.0092	2.801	0.0373

*Ccl2*†	20296	chemokine (C-C motif) ligand 2; MCP-1	2.263	0.021	3.325	0.00493

***Ccl4*†**	20303	chemokine (C-C motif) ligand 4; MIP-1B	4.232	0.0154	7.864	0.00302

*Ccl7*	20306	chemokine (C-C motif) ligand 7; MCP-3	3.183	0.00247	5.009	0.000346

***Ccl8*†**	20307	chemokine (C-C motif) ligand 8; MCP-2	3.12	0.00645	3.911	0.000657

*Ccl19*	24047	chemokine (C-C motif) ligand 19; ELC	2.119	0.00758	2.629	0.00228

*Ccl21a*	65956	chemokine (C-C motif) ligand 21; SLC	3.577	0.000615	2.66	0.0052

*Cx3cr1*	13051	Cx3cr1	2.602	0.00131	3.99	0.000356

*Ccr5*†	12774	CD195	2.224	0.00187	3.358	0.000184

**MMPs**						

***Mmp2*†**	17390	matrix metalloproteinase 2	2.513	0.000969	3.123	0.000609

*Mmp12*	17381	matrix metalloproteinase 12	2.172	0.0232	7.993	0.00031

*Mmp13*	17386	matrix metalloproteinase 13	6.428	0.02	13.62	0.00657

*Mmp14*	17387	matrix metalloproteinase 14	2.345	0.00432	3.99	0.000764

**Cell lineage**						

*Cd14*†	12475	CD14 antigen	2.068	0.00045	2.549	4.29E-05

*Cd68*†	12514	CD68 antigen	2.907	0.00865	5.776	0.00162

*Csf3r*	12986	colony stimulating factor 3 receptor (granulocyte); CD114.	2.627	9.99E-05	2.53	0.00056

*FceR1g*†	14127	Fc receptor, IgE, high affinity I, gamma polypeptide; CD23	2.835	0.0275	3.691	0.0125

*Anpep*	16790	alanyl (membrane) aminopeptidase; CD13	2.216	0.000594	3.312	5.46E-05

**Cytokines and receptors**						

***Il6***†	16193	interleukin 6	2.61	0.0411	3.92	0.0136

*Il1b*†	16176	interleukin 1 beta	3.966	0.0158	5.89	0.00501

**Antigen processing and presentation**						

*Cd74*	16149	Ia-associated invariant chain	2.376	0.00037	2.488	7.03E-05

*H2-Ab1*†	14961	histocompatibility 2, class II antigen A, beta 1	2.799	0.000852	3.108	5.10E-06

Shown are genes which were significantly up-regulated (2-fold, uncorrected p value < 0.05 calculated with unpaired t-test) within the aortas of mice with AAAs by comparison to both mice that received angiotensin II and did not develop AAAs and also saline control mice. Genes were selected from the full list of differentially expressed genes (shown in Additional Files 3 and 4) based on their upregulation in both comparisons, likely functional importance and previous association with human AAA. Transcripts associated with no recognised gene are not included. Genes highlighted with † have previously been identified as associated with human AAA in a whole genome expression study or other human AAA studies [9,10]. Genes highlighted in bold were selected for validation by real time PCR (see Table 6). Cf: compared to; ns: not significant

**Table 5:** Examples of genes upregulated in the aortas of mice exposed to angiotensin II which did not develop aneurysms. These genes are potentially protective against AAA.

**Gene symbol**	**Gene ID**	**Description**	**Fold increase cf AAA**	**P value**	**Fold increase cf saline**	**P value**
*Cald1*†	*109624*	caldesmon 1	2.053388	0.000625	2.379	5.64E-06

*Acta2*†	*11475*	actin, alpha 2, smooth muscle, aorta	2.178649	0.0284	2.536	1.84E-06

*Sost*†	*74499*	sclerostin	2.659574	0.0118	2.167	9.01E-06

*Kcnmb1*†	*16533*	potassium large conductance calcium-activated channel, subfamily M, beta member 1.	2.004008	0.0129	2.262	7.45E-07

*Dstn*†	*56431*	destrin	2.169197	0.00498	2.242	6.70E-06

*Timp4*†	*110595*	tissue inhibitor of metalloproteinase 4	2	1.99E-05	ns	ns

*Pdlim3*†	*53318*	PDZ and LIM domain 3	2.12766	0.00386	ns	ns

*Rgs17*†	*56533*	regulator of G-protein signaling 17	2.439024	0.00106	ns	ns

*Hspa1l*	*15482*	heat shock protein 1-like	2.020202	0.00844	ns	ns

*Gabra3*	*14396*	gamma-aminobutyric acid (GABA-A) receptor, subunit alpha 3	2.028398	0.00482	ns	ns

*Btc*†	*12223*	betacellulin, epidermal growth factor family member	2.314815	0.0412	ns	ns

*Fbxo30*	*71865*	F-box protein 30	2.325581	0.000911	ns	ns

*Hspa1*†*a*	*193740*	heat shock protein 1A	2.262443	0.00944	ns	ns

*Mtap1b*†	*17755*	microtubule-associated protein 1B	2.252252	0.0363	ns	ns

*Xirp1*	*22437*	cardiomyopathy associated 1	2.155172	0.0165	ns	ns

*Klk10*	*69540*	Kallikrein 10	2.136752	7.42E-05	ns	ns

*Slc22a1*†	*20517*	solute carrier family 22 (organic cation transporter), member 1	2.873563	0.000794	ns	ns

Shown are examples of genes which were significantly up-regulated (2-fold, uncorrected p value < 0.05 calculated with unpaired t-test) within the aortas of mice that received angiotensin II and did not develop AAAs by comparison to mice which developed AAAs. Also shown is comparison with findings from the aortas of saline control mice. Genes were selected from the full list of differentially expressed genes (shown in Additional File 9) based on their likely functional importance and previous association with human AAA. Genes highlighted with † have previously been identified as down-regulated within human AAA in a whole genome expression study [9]. Cf: compared to; ns: not significant.

**Figure 2:**
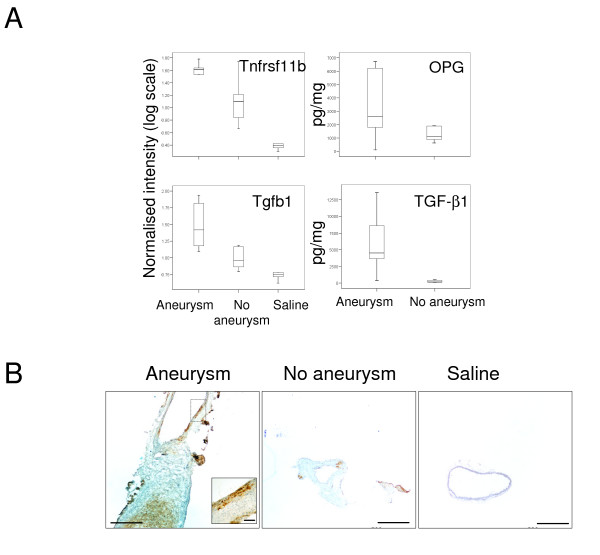
**Verification of microarray data for two genes in which aortic expression was related to aneurysm formation**. A) ELISA for aortic osteoprotegerin and transforming growth factor beta-1 (right panel) confirmed microarray expression data (left panel). Gene expression data are presented as boxplots of normalised intensity for *Tnfsf11b *(OPG) and *Tgfb1 *(TGFb-1) for each group (aneurysmal mice n = 5, non-aneurysmal mice n = 7, saline controls n = 6). ELISA data from suprarenal AAAs (n = 9) and suprarenal aortas without AAA (n = 9) of ApoE^-/- ^mice infused with angiotensin II. B) Immunohistochemistry confirms OPG localisation to aortic media (inset) and thrombus in suprarenal aortas with aneurysm. Aneurysm resistant and saline control aortas showed minimal OPG staining. Scale bars represent 500 μm, except inset where bar represents 50 μm.

Based on previous studies demonstrating the importance of inflammation in AAA we selected a number of inflammation associated markers to validate which were differentially expressed between aortic aneurysms and control aortas [9]. Initially we confirmed the up-regulation of *Il6*, *Ccl4*, *Ccl8 *and *MMP2 *within aortic aneurysms using real time PCR (Table 6). These genes were highly upregulated in aortic aneurysms by comparison to aortas from both control groups in microarray studies and real time PCR (see Tables 4 and 6). We also confirmed the differential expression of the *Tnfrsf11b *(osteoprotegerin) and *Tgfb1 *(TGF-b1) genes across the three groups translated to differences in protein expression using samples from study 3. We measured the concentration of OPG and TGFb-1 in the suprarenal segments of angiotensin II treated mice that developed AAA (mean maximum diameter 2.67 ± 0.24, n = 9) and those that did not (mean maximum diameter 1.26 ± 0.25, n = 9, p < 0.01) and found that both proteins were expressed at higher concentrations in aortas of mice with aneurysms, p < 0.05 (Figure 2A). Using immunohistochemistry we demonstrated that OPG was found within the medial layers and remodeled adventitia of aortas with aneurysm, whereas little was detected in aneurysm-resistant aortas or saline controls (Figure 2B).

**Table 6:** Validation of genes up-regulated in AAA by real time PCR.

**Gene symbol**	**Gene ID**	**Description; synonym**	**Relative expression in AAA**	**Relative expression in no AAA**	**Relative expression in saline control**	**P value AAA v no AAA**	**P value AAA v saline control**
*Ccl4*†	20303	chemokine (C-C motif) ligand 4; MIP-1B	52.71 (19.80–133.40)	5.55 (4.87–9.91)	1.40 (0.84–2.49)	0.009	0.004

*Ccl8*†	20307	chemokine (C-C motif) ligand 8; MCP-2	8.84 (5.94–12.25)	1.66 (1.27–2.73)	0.84 (0.55–1.18)	0.009	0.004

*Mmp2*†	17390	matrix metalloproteinase 2	4.58 (3.80–11.00)	2.30 (1.50–3.17)	0.97 (0.81–1.06)	0.004	0.004

*Il6*†	16193	interleukin 6	70.87 (14.40–184.59)	9.49 (2.54–16.83)	0.46 (0.17–1.26)	0.030	0.004

Shown are median (inter-quartile range) of relative gene expression (compared to house-keeping gene) in whole aortas used in the micro-array studies removed from mice exposed to angiotensin II that developed AAAs (n = 5) or no AAA (n = 7) or saline controls (n = 6).

### Investigation of known functional KEGG pathways related to the differentially expressed genes

**Figure 3:**
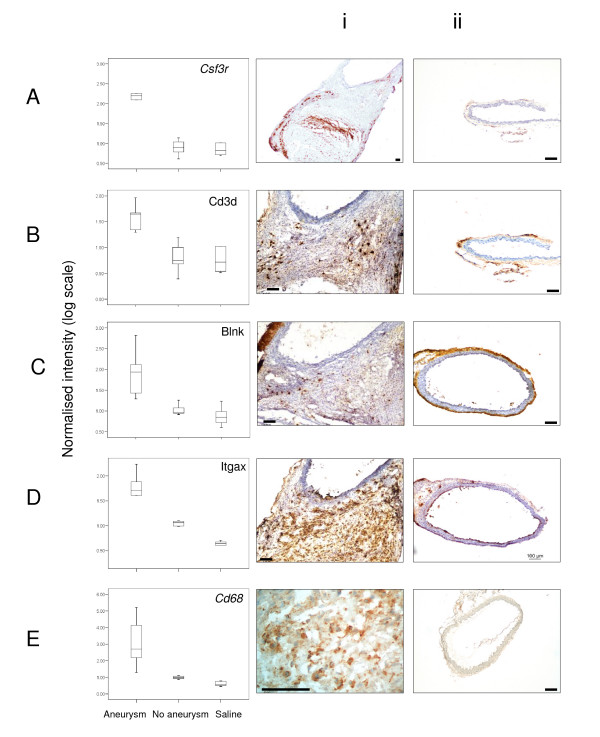
**Immunohistochemistry demonstrating inflammatory cells in the suprarenal aorta of mice with aneurysms demonstrating functional confirmation of microarray expression data**. Boxplots show normalised expression of genes that are markers of various haematopoietic cell lineages; *Csf3r *(neutrophils), *Cd3d *(T lymphocytes), *Blnk *(B lymphocytes), *Itgax *(dendritic cells) and *Cd68 *(monocyte/macrophages) from aneurysmal (n = 5), non-aneurysmal (n = 7) and saline control (n = 6) mice. Shown are sections stained for (A) neutrophils, (B) T lymphocytes (C) B lymphocytes (D) dendritic cells (E), macrophages in mice with aneurysms (i) and those without aortic dilatation (ii). Scale bars represent 100 μm.

KEGG pathway analysis of transcripts upregulated in the AAA group compared to the no AAA group using Webgestalt, revealed that of the 302 known genes in the input list, 116 of these could be assigned to one or more KEGG pathways (Additional File 10). This pathway analysis revealed a significant enrichment for genes involved in leukocyte recruitment, transendothelial migration and B lymphocyte receptor signalling and identified a number of cell lineage specific genes associated with aneurysm (Table 7). Chemokine genes upregulated in aneurysms included Ccl21 and Ccl19 (which attract T cells and dendritic cells), Ccl7 (attracts monocytes), Cxcl9 (attracts T cells), Cxcl12 (attracts lymphocytes) and Cxcl5 (attracts neutrophils), suggesting that all these inflammatory cell types are recruited to mouse aortic aneurysms. Genes that can be considered as markers for the following cell types were upregulated in aneurysms (Additional Files 5 and 6): neutrophils (Csf3r); T lymphocytes (Cd3d) and B lymphocytes (Blnk, Cd72, various immunoglobulin genes); and monocyte/macrophages (Cd14, Cd68). We used IHC to confirm the recruitment of neutrophils, T and B lymphocytes, dendritic cells and monocyte/macrophages to aneurysmal but not aneurysm-resistant aortas (Figure 3).

**Table 7:** Top 10 KEGG pathways enriched in mouse aortic aneurysms.

**Pathway (KEGG linked)**	**Total genes in pathway**	**Number genes upregulated in pathway in AAA v no AAA**	**Representative Genes (Entrez Gene ID)**	P value*
Cytokine-cytokine receptor interaction	249	28	Cxcl5 (20311), Cxcl9 (17329), Cxcl10 (15945), Cxcl12 (20315), Cxcl13 (55985), Cxcl16 (66102), Cxcl14 (57266), Xcl1(16963), Cx3cr1 (13051), Cxcr4 (12767), Ccl19(24047), Ccl21a (20298), Ccl21b (18829), Ccl2 (20296), Ccl12 (20293), Ccl4 (20303), Ccl7 (20306), Ccl5 (20304), Ccl8 (20307), Ccr5 (12774), Il6 (16193), Csf3r (12986), Csf2rb2 (12984), Il7r (16197), Il2rg (16186), Il10ra (16154), Tnfrsf13b (57916), Il1b (16176)	*8.70e-26*

Leukocyte transendothelial migration	115	15	Cldn11 (18417), Mmp2 (17390), Mmp9 (17395), Ptpn11, Cybb (13058), Ncf2 (17970), Ncf4 (17972), Thy1 (21838), Cxcl12 (20315), Cxcr4 (12767), Rac2 (19354), Vav1 (22324), Ptk2b(19229), Cxcl13 (55985), Cxcl14 (57266)	*4.20e-15*

Focal adhesion	194	11	Col3a1 (12825), Col1a1 (12842), Comp (12845), Reln (19699), Thbs1 (21825), Thbs2 (21826), Igf1 (16000), Igf1r (16000), Vav1 (22324), Rac2 (19354), Itga11(319480), Itga6 (16403)	*1.22e-7*

B cell receptor signaling pathway	69	10	Cd72(12517), Cd79b (15985), Fcgr2b (14130), Inpp5d(16331), Btk (12229), Blnk (17060), Vav1(22324), Rac2(19354), Rasgrp3(240168), Ifitm1(68713)	*3.75e-11*

Natural killer cell mediated cytotoxicity	129	10	Hcst (23900), Klrd1 (16643), Fcgr3(14131), Fcer1g (14127), Tyrobp (22177, Ptk2b (19229), Vav1 (22324), Lcp2(16822), Cd48 (12506), Rac2 (19354)	*8.70e-9*

Hematopoietic cell lineage	86	10	H2-Eb1(14969), Il7r (16197), Cd3d (12500), Fcgr1(14129), Anpep (16790), Il6 (16193), Il1b(16176), Csf3r (12986), Itga6(16403), Cd14(12475)	*3.24e-10*

Jak-STAT signaling pathway	155	8	Il6(16193), Csfrb2(12984), Csf3r(12986), Il10ra(16154), Il2rg(16186), Il7r(16197), Jak3 (16453), Socs3(12702)	*1.18e-6*

ECM-receptor interaction	81	8	Reln(19699), Itga11(319480), Itga6(16403), Col3a1(12825), Col1a1(12842), Thbs1(21825), Thbs2(21826), Sdc3(20970)	*1.43e-7*

Toll-like receptor signaling pathway	101	7	Cd14(12475), Il6 (16193), Il1b(16176), Ccl5(20304), Ccl4(20303), Cxcl10(15945), Cxcl9(17329),	*3.52e-6*

Cell adhesion molecules (CAMs)	158	7	H2-Ab1(14961), H2-Eb1(14969), H2-DMb1(14999), Selpl (20345), Sdc3(20970), Itga6(16403), Cldn11(18417)	4.42e-5

Pathways were identified using WebGestalt by comparison of differentially expressed genes in the aortas of mice that did and did not develop aneurysms following angiotensin II infusion. Transcripts with >2 fold increase in expression in AAA compared to no AAA and p < 0.05 were submitted for pathway analysis. Pathways are linked to the KEGG website  and genes are linked to gene information. P value indicates the uncorrected significance of enrichment calculated from the hypergeometric test.

## Discussion

Mouse models of human diseases are potentially important tools with which to investigate mechanisms involved in the pathology and identify new treatments. Currently a number of animal models of human AAA are available [4]. Many of these models require significant interventions such as exposing the aorta and subsequently infusing or painting elastolytic solution on a segment of the artery to induce weakening and inflammation. Currently the angiotensin II infusion model is most commonly used, possibly due to the relatively ease with which aneurysms are produced and the lack of requirement for what might be considered artificial manipulation of the aorta. To our knowledge this is the first study to examine whole genome expression in relation to aneurysm formation within this model. Our findings demonstrate that aneurysms forming in ApoE^-/- ^mice have marked influx by a range of inflammatory cells and upregulation of cytokines which have all been previously demonstrated in human AAA [8,9,13-17]. These findings confirm those from more selective previous studies within this model [5-7,18-22]. Importantly they suggest the value of this model for investigating the inflammatory and cytokine aspects of human AAA. We also demonstrated the upregulation of a range of chemokines, cytokines and proteolytic enzymes, such as Ccl4, Ccl8, Il6 and Mmp2 which have been previously implicated in human AAA [9,10,23,24]. The upregulation of the latter genes in AAAs was validated using real time PCR. We identified genes up or downregulated in the aortas of mice resistant to aneurysm formation suggesting potentially protective and pathological roles of these genes respectively in AAA. Sclerostin expression is decreased within human AAA biopsies compared to controls, however the significance of this finding is unclear [9]. We found increased expression of the sclerostin gene (Sost) in the aortas of mice resistant to aneurysm formation compared to both mice with aneurysms and saline controls, suggesting that sclerostin may play a role in inhibiting aortic dilatation. The ability of sclerostin to antagonise transforming growth factor beta, which has been linked to aortic aneurysm development, could be of significance in an aortic protection role for this gene [25]. Acta2 (vascular smooth muscle actin) expression was increased in aortas resistant to aneurysm formation compared to other groups. Acta2 encodes the single most abundant protein in vascular smooth muscle cells, and mutations in the human gene are associated with ascending thoracic aortic aneurysms and dissections [26]. Increased expression of Acta2 and other genes encoding smooth muscle cell proteins (Cald1, Dstn), in aortas protected from aneurysm, highlight the importance of vascular smooth muscle cells in maintaining vascular integrity. We observed downregulation of the genes encoding kininogen (kng1), Apolipoprotein CI (Apoc1) and the neutrophil-associated leucine-rich alpha-2-glycoprotein 1 (Lrg1) amongst others, in aortas of mice resistant to aneurysm suggesting that they may be pathological genes. The role of these in aneurysm formation warrants further investigation.

We also investigated genes that might underlie the predilection for suprarenal aneurysm formation within this model, highlighting downregulation of a number of genes which may explain the susceptibility of the suprarenal aorta to aneurysm formation. Urocortin has been shown *in vitro *and within animal models to inhibit the effects of the renin-angiotensin system, thus downregulation of Ucn3 (urocortin 3) within the suprarenal aorta of ApoE-/- mice may be relevant to its predilection for aneurysm formation following angiotensin II infusion [11,12]. We also demonstrated downregulation of Lama3 (laminin alpha 3) within the suprarenal aorta. This extracellular matrix protein has been demonstrated to be present in reduced concentrations within some human AAAs and therefore may also be relevant to the preponderance of the suprarenal aorta to aneurysm formation [27,28]. *In vitro *laminin also plays important roles in modulating inflammation and MMP production suggesting its relevance to aneurysm formation [29,30]. Further studies will be required to investigate these candidate genes for example employing deficient mice.

A small number of previous studies have used expression arrays to examine gene transcripts associated with AAA in rodent models or patient samples [9,28,31-34] (Table 8). There are a number of issues to consider when interpreting the results of the present study and those previously conducted, such as sample sizes, controls used, model employed (in the case of animal studies) and adjustment for multiple testing. Only one of the previous studies was a whole genome expression assessment, similar to the current study, with other studies examining much smaller numbers of transcripts. The sample sizes employed in all studies means that the power to detect differentially expressed genes is limited; therefore all current studies would have been expected to generate a large number of false negatives. We have therefore concentrated on identifying genes which were identified in the current study and also in these previous investigations and highlighted these throughout our tables (a summary of our findings in comparison with previous studies is provided in table 8). A number of chemokines were identified in all studies underlining the findings of the current investigation. Other common mechanisms identified to be associated with AAA included pro-inflammatory cytokines and matrix proteolysis. The design of the current experiment has some advantages over previous studies in the use of controls employed. We utilised two control sample types within study 2, i.e. aortas from mice receiving saline infusion but importantly also from mice that received angiotensin II and did not develop aneurysms. This has enabled us to try and isolate effects associated with AAA rather than just those due to angiotensin II. Findings in previous rodent studies may have related in part simply due to the effects of elastase [32,33]. Controls for human expression array studies are problematic for a number of reasons, including the large amount of patient to patient variations, and the effects of factors that are impossible to control for in small samples such as differences in medication taken and co-morbidities [9,28,34].

**Table 8:** Comparison of results from this study and those from other expression arrays for AAA.

**Ref.**	**Transcripts examined**	**Species**	**Number of samples/controls**	**Tissue of interest**	**Control tissue**	**Similar genes upregulated**
9	18057 oligonucleotides microarray	Human	6/7	AAA	Normal#	Chemokines (e.g. CCL2, CCL4, CCL8, CCR5, CXCL5, CXCR4, TNFRSF13B) Pro-inflammatory cytokines (e.g. IL1B, IL2RG) Matrix metalloproteinase (e.g. MMP9) Cell lineage markers (e.g. CD53, CD68, CD72)

32‡	1181 cDNA clones	Mice	3/3**	Elastase induced AAA	Inactivated elastase infused aortas	Pro-inflammatory cytokines (e.g. IL1B, IL6, INF, IGF1) Chemokines (e.g. CCL7) JAK-STAT signalling (e.g. SOC3)

31‡	375 cDNA clones	Human	1/1*	AAA body	AAA neck	Chemokines (e.g. CXCR2)

28‡	265 cDNA clones	Human	7/5/5†	AAA	AOD and normal†	Matrix metalloproteinase (e.g. MMP9, MMP12) Pro-inflammatory cytokines (e.g. IL1B, IL6) Chemokines (e.g. CCL4, CCR5) Fibrinolysis (e.g. uPA)

33‡	8799 cDNA clones microarray	Rat	1/1‡‡	Elastase induced AAA	Saline infused aortas	Oxidative stress (Heme oxygenase, lipoxygenase)

*34‡*	1176 cDNA clones	Human	4/4	AAA	normal††	Cathepsins (e.g. cathepsin H) Matrix metalloproteinase (e.g. MMP9) Chemokines (e.g. CXCR4, CCL5)

*Results provided limited; †5 controls from aortic occlusive disease (AOD) from patients undergoing bypass and 5 controls from organ donors which were not age-matched; ‡These studies did not adjust for multiple testing; **pooling of samples was performed with 3 pools of interest compared with 3 controls for 4 different time periods after elastase infusion; ††Controls were from organ donor who were not matched; ‡‡Appears to have been comparison of one pool of samples from elastase induced aaas and one pool from controls examined on two microarrays each; #controls from post-mortem samples. Ref. = reference.

This study is the first to carry out a whole genome expression analysis within the angiotensin II- Apo E^-/- ^model. We used an explorative approach using relatively small numbers of arrays (28 in total were included in the array studies). We were not adequately powered to adjust for multiple testing given the number of transcripts examined. Unlike some other microarray studies we have used real time PCR, IHC and ELISA to confirm some of our findings rather than relying on RNA assessment alone [9]. Numerous studies have now demonstrated the validity of microarray platforms therefore we felt it would be useful to demonstrate the functional relevance of altered RNA expression [9,35]. Our findings suggest the value of this mouse model as one to investigate the role of inflammation, cell recruitment and proteolysis in AAA.

## Conclusion

In conclusion our study supports the value of the angiotensin II infused ApoE^-/- ^mouse model for investigating mechanisms and interventions relevant to human AAA.

## Methods

### Study design

Infusion of angiotensin II induces aortic dilatation particularly affecting the suprarenal aorta in ApoE^-/- ^mice [5,6,18-21]. Based on studies carried out in our and other laboratories the response to angiotensin II is variable, with some mice developing large aneurysms but other animals appearing resistant to aneurysm formation with aortic diameters similar to that of saline controls (Additional file 12). The infrarenal aorta is protected from aneurysm formation in these mice. To assess the likely signalling pathways relevant to aneurysm development and progression within this mouse model we carried out three studies (Table 1): 1) We compared RNA expression within segments of supra and infrarenal aortas from 13 week old male ApoE^-/- ^mice unexposed to angiotensin II (n = 10). 2) We compared RNA expression from whole aortas of 17 week old male ApoE^-/- ^mice which had been exposed to angiotensin II (1.44 μg/kg/min) for 4 weeks where there was clear evidence of aortic aneurysm formation (n = 5) with that of mice failing to develop aneurysms (n = 7) and those exposed to saline infusion (n = 6). 3) We selected 2 genes and 5 cellular pathways identified as upregulated within the aortas of mice with aneurysms for validation using protein assessments including ELISAs and immunohistochemistry (IHC). The selection of genes was based on their association with pathways we had identified from pathway analysis of the data and recognised ability to be able to assess them using ELISAs or IHC. For study 3, twenty-eight additional 17 week old male ApoE^-/- ^mice were infused with angiotensin II for 4 weeks. Ten mice in study 3 died prematurely due to aortic rupture and were excluded from further analysis in order to avoid post-mortem or post rupture effects confounding assessments, leaving 18 animals in this group.

### Mice

This investigation conformed to the Guide for the Care and Use of Laboratory Animals published by the US National Institutes of Health (NIH Publication No. 85-23, revised 1996). Ethical approval was obtained from the local institutional committee prior to commencement of the study. Male ApoE^-/- ^mice were obtained from Animal Resources Centre, Canning Vale, Western Australia aged 6–8 weeks. Mice were fed standard chow, and at 13 weeks either their aortas were harvested (study 1, n = 10) or angiotensin II (n = 12 study 2; n = 28 study 3) or saline (n = 6 study 2) infusion commenced. Aortic harvesting and osmotic minipump placement (Model 2004, ALZET, Durect Corporation, Cupertino, California, USA) was carried out under ketamine (150 mg/kg i.p.) and xylazine (10 mg/kg i.p.) anaesthesia as previously described [22]. The 28 day infusion period and dose of angiotensin II were selected as these have been routinely used in other studies within this model [5,6,18-21]. In studies 1 and 2, mice were euthanized by carbon dioxide asphyxiation, the aortas perfused with RNAlater^® ^(Qiagen, Doncaster, Victoria, Australia), and harvested from arch to iliac bifurcation and stored at -80°C for later analysis. For study 3 mice were euthanized, aortas were perfused with PBS and the suprarenal aortic segments divided into 2 and stored separately at -20°C for later IHC and cytokine assessment.

### Aorta Morphometry

Aortas were placed on a black background and digitally photographed (Coolpix 4500, Nikon). Maximum diameters of the aortic arch, thoracic, suprarenal and infrarenal aorta were determined from the images using computer-aided analysis (Scion Image, Scion Corporation). Preliminary studies (n = 27) established that these measurements could be repeated with good intra-observer reproducibility (Coefficient of repeatability 0.98, 95% confidence intervals 0.975–0.982, and coefficient of variation 4%).

### Microarrays

Total RNA was extracted from homogenised aortas using the TRI reagent (Sigma) and RNeasy Mini kits (Qiagen) and analysed on the HP 2100 Bioanalyzer (Agilent Technologies) for integrity using Eukaryotic total RNA nano chips (Agilent) (Additional File 11). All samples had RNA integrity scores between 6.9 and 8.1. For study 1, RNA extracted from the pooled suprarenal aortas of two mice was compared with that obtained from the infrarenal aortic segments of the same mice. A total of 10 mice (i.e. 5 pairs of pooled segments) were included in study 1. For study 2, RNA extracted from the whole aortas of mice with aneurysms (n = 5, Additional File 12A) was compared with that extracted from the whole of aortas of saline controls (n = 6, Additional File 12C) and mice exposed to angiotensin II which did not develop aneurysms (n = 7, Additional File 12B). No sample pooling was carried out in study 2. RNA hybridization was performed, and gene expression profiles determined, using CodeLink Mouse Whole Genome Bioarray chips (GE healthcare, Amersham, Bioscience) (study 1, n = 10) and Illumina Mouse Sentrix 6 version 1.1 Beadchips (study 2, n = 18). For study 1 total RNA from infrarenal and suprarenal aortas was supplied to GenUS BioSystems Inc. (Northbrook, Illinois USA) who conducted cDNA synthesis, hybridization and scanning using standard protocols. In brief, 10 μg of total RNA was used for each 'sample versus sample' comparison and first and second strand cDNA were prepared using the Codelink™ iExpress Expression assay Reagent kit (GE healthcare Amersham). Biotinylated cRNA target was prepared from the cDNA template by linear amplification using biotin-dNTPs and verified on a Bioanalyzer 2100. The cRNA was fragmented to uniform size and once again verified on the Bioanalyzer 2100. Each fragmented, biotin-labeled cRNA was added to a Codelink Mouse Whole Genome array which contained 34,957 probes targeting unique mouse transcripts. The bioarrays were washed, exposed to Cy5 streptavidin and scanned using GenePix 4000B laser scanner. Scanned image files were examined using CodeLink image and data analysis software (GE healthcare, Amersham, Bioscience). The image information was converted into spot intensity values using CodeLink™ Expression Analysis software (GE healthcare). The generated values were exported to GeneSpring GX 7.3.1 software (Silicon Genetic, USA) for further analysis. Study 2 labelling, hybridisation and scanning was done at the SRC Microarray Facility, University of Queensland, Brisbane, Australia according to the manufacturer's instructions. For study 2 total RNA was amplified in a single-round of *in vitro *transcription amplification that allowed incorporation of biotin-labeled nucleotides using the Illumina TotalPrep RNA amplification kit (Ambion, Inc., Austin, TX). mRNA samples were assessed for integrity and purity prior to hybridization using the Bioanalyser 2100 with mRNA Nano chips (Agilent Technologies). cRNA of each sample was hybridized to an Illumina Mouse WG-6 V1.1 BeadChip followed by washing, blocking, and streptavidin-Cy3 staining steps, and scanning with a high-resolution Illumina BeadArray reader scanner. The data extraction was performed by using Illumina Bead Studio V2.3.41 software with the output being raw, non-normalized bead summary values. The BeadStudio matrix contains the summarised expression values (Avg_Signal), standard error of the bead replicates (BEADSTDEV), number of beads used (Avg_NBEADS) and a detection score, which estimates the confidence limit of detection of a gene. The performance of the built-in controls that accompany each Illumina beadchip experiment was assessed as part of the Bead Studio V2 experiment performance report and was found to be satisfactory. Controls included Housekeeping controls for intactness of the biological specimens, negative controls to establish gene expression detection limits and hybridization controls including low and high stringency controls and biotin signal generation controls. The raw data matrix extracted from Beadstudio was uploaded into GeneSpring GX 7.3.1 (Silicon Genetics, Redwood City, CA) software for downstream analysis. Details of GeneSpring analysis including data transformations, per chip and per gene normalisations are described below in Analysis and Design.

### Real time PCR

Using RNA obtained for the mice employed in the micro-array investigations in study 2 we validated findings for 4 genes (*Ccl4*, *Ccl8*, *Il6 *and *Mmp2*) using real time PCR. RNA samples from mice exposed to angiotensin II that developed aneurysms (n = 5), mice exposed to angiotensin II that did not develop aneurysms (n = 7) and saline control mice (n = 6) were included in the analysis. The QuantiTect SYBR Green one-step RT-PCR Kit (Qiagen) and Quantitect Primer Assays (Qiagen) (*Il6*, QT00098875; *Ccl4*, QT00154616; *Ccl8*, QT00128548; *Mmp2*, QT00116116) were used according to the manufacturer's instructions with 25 ng of total RNA as template. Primers for mouse *Gapdh *were used to amplify the housekeeping gene (Qiagen, QT01658692). Standard curves for each gene including the housekeeping gene were constructed using duplicate sets of five (5) 10-fold serial dilutions of equal volumes of the pooled saline control RNAs. Negative and "minus-RT" controls were also included. All reactions were independently repeated in duplicate to ensure the reproducibility of the results. Cycling parameters were as follows: 50°C 30 min for RT; 95°C 15 min; 40 cycles of 94°C 15 sec, 55°C 30 sec, 72°C 30 sec. Data were viewed and analysed using the Rotor-Gene's real-time analysis software (Rotor-Gene 6000; Corbett Life Science, Sydney). The relative expression of the gene of interest in each sample was calculated by the Rotor-Gene software using the concentration-Ct-standard curve method and normalised using the average expression of *Gapdh *for each sample. SSPS statistical software was used to calculate median and interquartile ranges for expression of each gene of interest in each group.

### IHC

Serial cryostat sections 7 μm thick were cut from suprarenal aortas with and without aneurysms prior to staining for inflammatory cells. Serial frozen sections were air-dried, fixed in acetone for 10 min at -20°C, air dried and rehydrated with PBS before being incubated in 3% H_2_O_2_/0.1% sodium azide/PBS to block endogenous peroxidase. For macrophage detection, sections were blocked in 2% normal goat serum in PBS followed by staining using pan-macrophage antibody (clone MOMA-2, Abcam AB33451), and goat anti-rat HRP (Millipore AP136P). Rat IgG (Sigma I4131) was used as isotype control. Detection of osteoprotegerin (OPG) was carried out using biotinylated goat anti-mouse OPG (R&D BAF459) diluted to 1 μg/ml in blocking buffer followed by Vectastain Elite ABC-HRP (Vector Laboratories). Biotinylated goat-IgG was used as isotype control (Vector Laboratories VEBI1001). T cells (CD3e FITC clone 145-2C11, BD Biosciences 553061), B cells (CD45R/B220 biotin clone RA3-6B2, BD Biosciences 550385), dendritic cells (CD11c biotin clone HL3, BD Biosciences 553800) and neutrophils (Ly6G/Ly6C(Gr-1) FITC clone RB6-8C5, BD Biosciences 553127) were demonstrated using either anti-FITC HRP (Invitrogen), biotinyl-tyramide (Perkin Elmer), SA-HRP (Perkin Elmer) for FITC-conjugated antibodies or SA-HRP, biotinyl-tyramide, SA-HRP for biotinylated antibodies. Appropriate isotype controls were added to other sections (all BD Biosciences). Slides were incubated in the peroxidase substrate 3, 3'-diamminobenzidine (ImmPACT DAB, Vector), counterstained in Mayer's Haematoxylin, dehydrated, cleared in xylene and mounted in Depex mounting medium. Sections were photographed using a Nikon Eclipse 50i microscope, Digital Sight camera and NIS-elements software.

### Cytokine studies

Protein was extracted from individual frozen suprarenal aortic segments by homogenising in buffer (10 mM cacodylic acid, 60 mM L-arginine, 0.25% triton x-100 in PBS, pH 7.2) and centrifuging at 18,000 × *g *at 4°C for 20 min. Supernatant protein was quantified by the Bradford technique (Protein Assay, Bio-Rad, Hercules, California, USA). Concentrations of OPG and transforming growth factor beta-1 (TGFb-1) were assessed using commercial ELISAs (Quantikine, R&D Systems, MOP00 for OPG, MB100B for TGFb-1) and expressed as pg/mg of protein. Initial tests were performed to determine an appropriate amount of extracted aortic protein to load in each well to suit the ELISA standard curves (10 μg for OPG ELISA, 20 μg for TGFb-1 ELISA). Serum and extracted aortic proteins from OPG deficient mice were confirmed as having zero readings using the OPG ELISA (data not shown). Batch analysis of the samples was carried out to facilitate comparison between groups. We have previously reported excellent reproducibility of similar assays [36].

### Analysis and design

We designed the current study as an exploratory analysis expecting a minimal level of false discovery inevitable in analysis of large microarray gene probes. Sample size calculations were conducted using SPCalc [37,38]. For study 1 (which included paired samples) we aimed to detect a 1.5-fold difference at a power of 0.8 (alpha uncorrected 0.05), while for study 2 (which included unpaired samples) we aimed to detect a 2-fold difference. Based on the number of gene probes to be examined (26,522) we wished to control the false positives to a mean of 2 [17,18]. We estimated the total number of arrays needed for study 1 and 2 were 10 and 18 respectively. Microarray data were imported into Genespring GX 7.3.1 (Agilent) for analysis. The data from the individual bioarrays have been deposited in NCBIs Gene Expression Omnibus (GEO; ) and are accessible through GEO accession number GSE7006 (GEO Accession viewer) for study 1 and GSE12591  for study 2. For study 1 which used paired samples, normalisations were as follows: each chip was normalised to the median intensity of the array; each gene was normalised relative to the suprarenal sample of each pair. Thus the expression of each SRA sample was normalised to 1. Volcano plots were used to identify differentially expressed genes with >1.5 fold difference in expression and p < 0.05. Statistical analysis was carried out using a paired t-test (unequal variances) comparing the mean infrarenal/suprarenal ratio to a baseline value of 1 for each sample pair. In study 2 we followed the standard normalization procedures recommended for the GeneSpring GX 7.3.1 software for one-colour array data. In brief, data transformation was corrected for a low signal, with values recorded at <0.01 increased to the minimum (0.01). Default settings were used for experiment normalisation which included chip normalisation to 50th percentile and gene normalisation to the median. The cross gene error model was used to further filter low quality data. We sought to identify genes with a 2-fold differential expression within the aortas of mice with and without aneurysms based on an uncorrected p value of < 0.05 included in our sample size estimate. We initially compared findings from mice that received angiotensin II and developed AAAs (n = 5) with both other groups of mice (n = 7 and 6). Genes showing a greater than 2 fold difference in expression between groups (unpaired t-test (unequal variance) p < 0.05), were considered to be differentially expressed. We reasoned that the most significant findings would relate to genes differentially expressed between angiotensin II perfused mice, that did (n = 5) and did not (n = 7) develop aneurysms. Volcano plots were used to identify differentially expressed genes between the aortas of these two groups of mice. This group of 531 genes was further investigated by hierarchical clustering in GeneSpring, in which a tree of transcripts or genes is built by successively finding the two most similar gene expression patterns from the full transcript set. Individual samples were then arranged in a condition tree according to overall similarity. We also included the saline group in this clustering analysis in order to show how these 531 genes are expressed in control aortas. Genes with similar expression patterns were grouped as hierarchical clusters with distances between samples computed using Pearson correlations for similarity measures and average linkage as the clustering algorithm. Hierachical clustering revealed several major nodes in the gene tree structure which indicated at least 4 different patterns of gene expression. Lists of differentially expressed genes were examined for biologically relevant associations using Gene Ontologies and Kyoto Encyclopedia of Genes and Genome (KEGG) pathway analysis in the web-based software Webgestalt . Quantitative real time PCR outcomes in study 2 and concentrations of cytokines measured in study 3 were compared between groups using Mann Whitney U test.

## Authors' contributions

CR carried out the expression arrays, analysis of results and immunohistochemistry, and participated in drafting the manuscript. MN carried out the mice work. AT carried out the real time PCR. BC carried out the ELISAs, analysed these results and participated in drafting the manuscript. JVM participated in drafting and editing the manuscript. JG conceived the study, obtained funding, supervised the project and wrote the main manuscript. All authors read and approved the final manuscript.

## Supplementary Material

Additional file 1 **List of transcripts differentially expressed in supra and infrarenal aortas**. Transcripts included were expressed at 1.5 fold different levels and significant at uncorrected p < 0.05. Included are genebank and unigene names, uncorrected p values and ratio of transcripts expression compared between supra and infrarenal aortas. Click here for file

Additional file 2 **Number of differentially expressed transcripts comparing aortas of mice with and without aneurysms, in addition to saline controls**. Transcripts included were expressed at 2 fold different levels and significant at uncorrected p < 0.05. Click here for file

Additional file 3 **List of transcripts differentially expressed in aortas of mice exposed to angiotensin II which did (AAA group) and did not (no AAA group) develop aneurysms (n = 531)**. We considered this the most important comparison since the controls were in all ways similar to the group of interest except they did not have AAAs. Transcripts included were expressed at 2 fold different levels and significant at uncorrected p < 0.05. Transcripts with higher expression in AAA group are indicated in yellow, whilst those with higher expression in no AAA group are coloured blue. Included are gene symbol (where an identified gene is associated with the transcript), genebank accession number, fold difference, uncorrected p values, gene description, and Illumina Probe identification number. Click here for file

Additional file 4 **List of transcripts differentially expressed in aortas of mice exposed to angiotensin II which developed aneurysms (AAA) and saline controls (n = 1196)**. Transcripts included were expressed at 2 fold different levels and significant at uncorrected p < 0.05. Included are gene symbol (where an identified gene is associated with the transcript), genebank accession number, fold difference, uncorrected p values, gene description, and Illumina Probe identification number. Click here for file

Additional file 5 **List of transcripts differentially expressed in aortas of mice exposed to angiotensin II which did not develop aneurysms (no AAA) and saline controls (n = 654)**. Transcripts included were expressed at 2 fold different levels and significant at uncorrected p < 0.05. Included are gene symbol (where an identified gene is associated with the transcript), genebank accession number, fold difference, uncorrected p values, gene description, and Illumina Probe identification number. Click here for file

Additional file 6 **List of pattern 1 transcripts (n = 104) which were downregulated with the aortas of aneurysm resistant mice (see results and Figure **1**)**. Included are gene symbol (where an identified gene is associated with the transcript), genebank accession number, uncorrected p values, gene description, Illumina Probe identification number and fold difference comparing aortas of mice exposed to angiotensin II that did (AAA) and did not develop aneurysms (no AAA). Click here for file

Additional file 7 **List of pattern 2 transcripts which were upregulated in aortic aneurysms (n = 325; see text and figure **1**)**. Included are gene symbol (where an identified gene is associated with the transcript), genebank accession number, uncorrected p values, gene description, Illumina Probe identification number and fold difference compared to aortas of mice with no aneurysm (no AAA). Click here for file

Additional file 8 **List of genes which were downregulated in the aortas of mice exposed to angiotensin II which did not develop aneurysms (no AAA) by comparison to both mice developing aneurysms (AAA) and also saline controls (n = 10)**. The list of genes was generated from pattern 3 transcripts excluding those that were associated with no known gene or which were not significantly different (2-fold, p < 0.05) by comparison to saline controls in addition (n = 7). These genes potentially have a pathological role in AAA. Genes highlighted with † have previously been identified as upregulated within human AAA biopsies in a whole genome expression study [9]. Included are gene symbols, gene description, fold difference and uncorrected p values comparing aortas with aneurysms and saline control aortas to aneurysm-resistant aortas. Click here for file

Additional file 9 **List of transcripts which were upregulated in the aortas of mice resistant to aneurysm formation (n = 85)**. Transcripts highlighted in yellow are increased (>2 fold, p < 0.05) in the no AAA group when compared to both AAA and saline groups. These genes are potentially protective role against AAA. Included are gene symbol (where an identified gene is associated with the transcript), genebank accession number, uncorrected p values, gene description, Illumina Probe identification number and fold differences when comparing aortas resistant to aneurysm formation to aortas with aneurysms and saline control aortas. Click here for file

Additional file 10 **Webgestalt analysis of transcripts upregulated in aortas of mice with aneurysms compared to those without**. Both groups of mice received angiotensin II. Of 376 recognised transcripts 302 were known genes and 116 were assigned to one or more KEGG pathways. Included are gene symbol (where an identified gene is associated with the transcript), gene description, Illumina Probe identification number and KEGG title. Click here for file

Additional file 11 **Examples of mouse RNA samples examined on the Agilent Bioanalyser**. The 10 pooled samples (2 supra or infra renal segments each) used in study 1 (with repetition of 1 sample) were analysed in comparison to molecular weight markers (far left), 18 and 28S RNA controls (far right). All samples had RNA integrity scores of between 7.5 and 8.1. Click here for file

Additional file 12 **Digital photographs of aortas from 17 week old male ApoE^-/- ^mice exposed to angiotensin II or saline subcutaneously for 4 weeks from study 2**. A) Example of an aorta from a mouse in which an aneurysm developed in response to angiotensin II infusion. B) Example of an aorta from a mouse in which an aneurysm did not develop in response to angiotensin II infusion. C) Example of an aorta from a mouse exposed to saline infusion for 4 weeks with no aneurysm formation. Click here for file
