# Supplementary material for: Whole genome expression analysis within the angiotensin II-apolipoprotein E deficient mouse model of abdominal aortic aneurysm
Source: BMC Genomics. 2009 Jul 6;10:298. doi: 10.1186/1471-2164-10-298 (PMC2728106; doi:10.1186/1471-2164-10-298)
Supplement: Additional file 2 — Number of differentially expressed transcripts comparing aortas of mice with and without aneurysms, in addition to saline controls. Transcripts included were expressed at 2 fold different levels and significant at uncorrected p < 0.05. [file 1471-2164-10-298-S2.pdf]

Supplementary Table 2. Number of differentially expressed genes (2 fold, p<0.05) in the aortas of mice with and without aneurysms and saline controls

Group	Aneurysm	No aneurysm	Saline controls
**Aneurysm**	-	531 (446 up, 85 down)	1196(749 up, 447 down)
**No aneurysm**	531 (446 up, 85 down)	-	654 (128 up, 526 down)
**Saline controls**	1196(749 up, 447 down)	654 (128 up, 526 down)	-
