# Supplementary material for: Whole genome expression analysis within the angiotensin II-apolipoprotein E deficient mouse model of abdominal aortic aneurysm
Source: BMC Genomics. 2009 Jul 6;10:298. doi: 10.1186/1471-2164-10-298 (PMC2728106; doi:10.1186/1471-2164-10-298)
Supplement: Additional file 8 — List of genes which were downregulated in the aortas of mice exposed to angiotensin II which did not develop aneurysms (no AAA) by comparison to both mice developing aneurysms (AAA) and also saline controls (n = 10). The list of genes was generated from pattern 3 transcripts excluding those that were associated with no known gene or which were not significantly different (2-fold, p < 0.05) by comparison to saline controls in addition (n = 7). These genes potentially have a pathological role in AAA. Genes highlighted with † have previously been identified as upregulated within human AAA biopsies in a whole genome expression study [9]. Included are gene symbols, gene description, fold difference and uncorrected p values comparing aortas with aneurysms and saline control aortas to aneurysm-resistant aortas. [file 1471-2164-10-298-S8.pdf]

Gene symbol	Gene ID	Description	Fold decrease cf AAA	P value	Fold decrease cf saline	P value
Kng1	*16644*	Kininogen 1	4.117	0.00109	4.184	9.47E-06
Apoc1†	*11812*	apolipoprotein C-I	3.408	0.00301	4.405	0.00017
Fgl1	*234199*	fibrinogen-like protein 1	2.182	0.0138	2.591	0.00112
Stxbp2†	*20911*	syntaxin binding protein 2	2.281	0.000228	2.624	3.32E-06
Lrg1	76905	leucine-rich alpha-2-glycoprotein 1	2.219	0.0142	2.583	0.00269
Gpx3	*14778*	glutathione peroxidase 3	2.597	0.000815	3.344	4.92E-06
Hp	*15439*	haptoglobin	2.275	0.001	2.857	0.00499
Chst1	*76969*	carbohydrate (keratan sulphate Gal-6) sulfotransferase 1	2.009	0.0255	2.439	0.000843
Eltd1	*170757*	EGF, latrophilin seven transmembrane domain containing 1	2.109	0.00424	2.336	5.48E-06
Robo4	*74144*	roundabout homolog 4	2.602	0.00527	2.618	2.76E-05
