# Supplementary figures and images for: Whole genome expression analysis within the angiotensin II-apolipoprotein E deficient mouse model of abdominal aortic aneurysm
Source: BMC Genomics. 2009 Jul 6;10:298. doi: 10.1186/1471-2164-10-298 (PMC2728106; doi:10.1186/1471-2164-10-298)

## Supplemental Figure 1

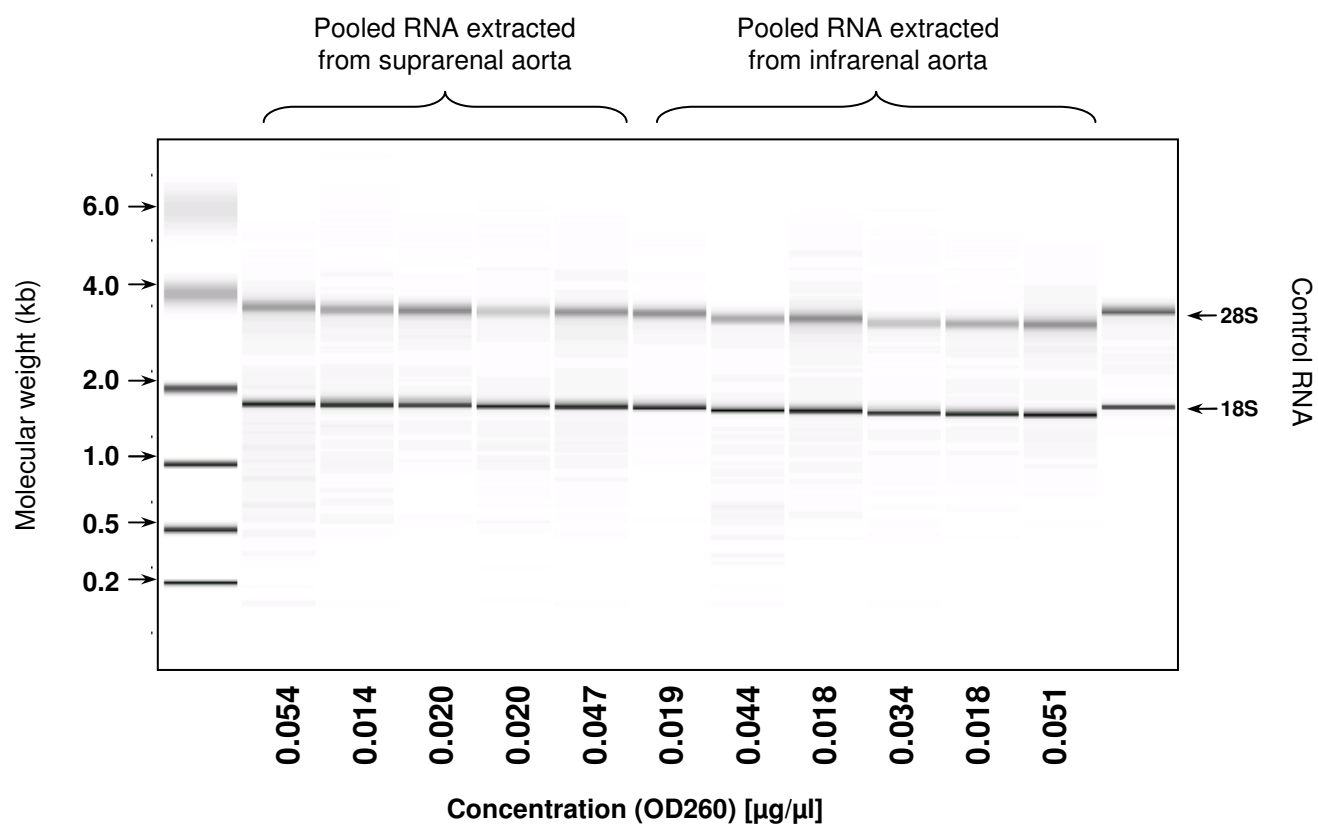

Supplement: Additional file 11 — Examples of mouse RNA samples examined on the Agilent Bioanalyser. The 10 pooled samples (2 supra or infra renal segments each) used in study 1 (with repetition of 1 sample) were analysed in comparison to molecular weight markers (far left), 18 and 28S RNA controls (far right). All samples had RNA integrity scores of between 7.5 and 8.1. [file 1471-2164-10-298-S11.pdf]

## Supplementary Figure 2

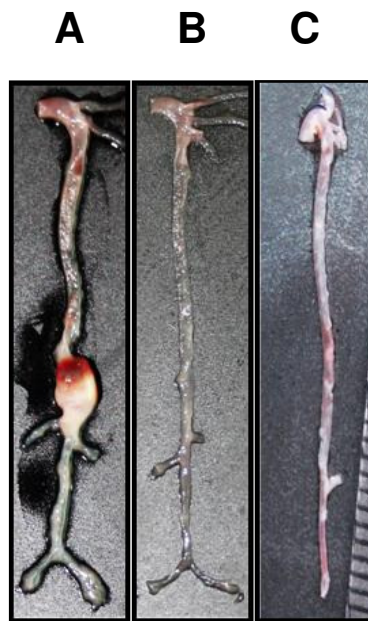

Supplement: Additional file 12 — Digital photographs of aortas from 17 week old male ApoE-/- mice exposed to angiotensin II or saline subcutaneously for 4 weeks from study 2. A) Example of an aorta from a mouse in which an aneurysm developed in response to angiotensin II infusion. B) Example of an aorta from a mouse in which an aneurysm did not develop in response to angiotensin II infusion. C) Example of an aorta from a mouse exposed to saline infusion for 4 weeks with no aneurysm formation. [file 1471-2164-10-298-S12.pdf]
